# Proteolytic Processing of Von Willebrand Factor by Adamts13 and Leukocyte Proteases

**DOI:** 10.4084/MJHID.2013.058

**Published:** 2013-09-02

**Authors:** Stefano Lancellotti, Maria Basso, Raimondo De Cristofaro

**Affiliations:** Institute of Internal Medicine and Geriatrics, Haemostasis and Thrombosis Center, Catholic University School of Medicine, ROMA – ITALY

## Abstract

ADAMTS13 is a 190 kDa zinc protease encoded by a gene located on chromosome 9q34. This protease specifically hydrolyzes von Willebrand factor (VWF) multimers, thus causing VWF size reduction. ADAMTS13 belongs to the A Disintegrin And Metalloprotease with ThromboSpondin type 1 repeats (ADAMTS) family, involved in proteolytic processing of many matrix proteins. ADAMTS13 consists of numerous domains including a metalloprotease domain, a disintegrin domain, several thrombospondin type 1 (TSP1) repeats, a cysteine-rich domain, a spacer domain and 2 CUB (Complement c1r/c1s, sea Urchin epidermal growth factor, and Bone morphogenetic protein) domains. ADAMTS13 cleaves a single peptide bond (Tyr1605-Met1606) in the central A2 domain of the VWF molecule. This proteolytic cleavage is essential to reduce the size of ultra-large VWF polymers, which, when exposed to high shear stress in the microcirculation, are prone to form with platelets clumps, which cause severe syndromes called thrombotic microangiopathies (TMAs). In this review, we a) discuss the current knowledge of structure-function aspects of ADAMTS13 and its involvement in the pathogenesis of TMAs, b) address the recent findings concerning proteolytic processing of VWF multimers by different proteases, such as the leukocyte-derived serine and metallo-proteases and c) indicate the direction of future investigations.

## Introduction

**Figure 1:**
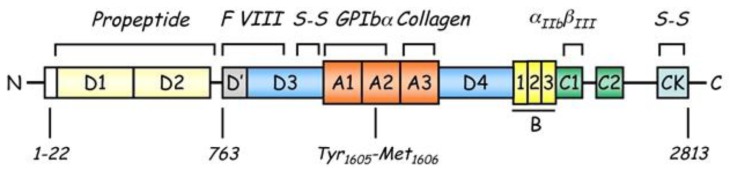
Scheme of von Willebrand factor monomer molecule with its functional domains. The prepro-VWF polypeptide is indicated with amino acids numbered from the amino- (aa 1) to carboxy-terminal portions (aa 2813). Binding sites are indicated for factor VIII (D′ and D3 domains), platelet glycoprotein Ibα (GPIbα) (A1 domain), collagen (A1 and A3 domains) and integrin αIIbβIII (RGDS sequence within the C1 domain). The cleavage site (Tyr1605-Met1606) for ADAMTS13 is located at the central A2 domain of von Willebrand factor. The locations of intersubunit disulfide bonds (S-S) are shown in the CK and D3 domains, which are important for the formation of VWF dimers and multimers, respectively.

The discovery of the metalloprotease referred to as ADAMTS13 (A Disintegrin-like And Metalloprotease with ThromboSpondin type 1 motif 13), as many other examples in biomedical research, found its way in the attempt to address the issue concerning the pathogenesis of severe forms of thrombotic microangiopathies (TMAs). The latter are a group of severe diseases characterized by deposition of blood platelet thrombi in the microcirculation, responsible for potentially fatal multi-organ failure. Moake et al.^1^ reported in 1982 the first evidence that the pathogenesis of the main form of microangiopathy, that is Thrombotic Thrombocytopenic Purpura (TTP), arises from a defect in proteolytic processing of von Willebrand factor (VWF), a multimeric glycoprotein with very high molecular weight that plays an essential role in platelet-dependent hemostasis. In 1996, 2 groups independently reported a metalloprotease that specifically cleaves VWF at the Tyr1605-Met1606 bond in the A2 domain.^2^,^3^ The proteolytic activity required VWF in a denatured conformation, achieved by preincubation with either low-concentration guanidine-HCl^3^ or urea,^2^ or by exposure to high shear stress *in vitro*.^3^ The proteolysis also required divalent cations such as Ba2+, Zn2+, Ca2+ or Co2+.^3^ A few years later, the protease was cloned, purified and characterized, and several groups identified the VWF-cleaving protease as ADAMTS13, a novel member of the ADAMTS family of metalloproteases.^4^–^8^ Considerable evidence now implicates the haemostatic protein VWF as a key component in TTP pathogenesis.^9^ VWF is an abundant plasma glycoprotein synthesized in all vascular endothelial cells and megakaryocytes as a precursor containing a signal peptide and large propeptide.^10^,^11^ Endothelial cell VWF is secreted via both constitutive and regulated pathways. In response to a variety of stimuli, VWF is released from endothelial cells as ultra-large (UL)-VWF, which can be up to approximately 20,000 kDa in size^12^,^13^ and are the most adhesive and reactive forms of VWF. UL-VWF form string-like structures attached to the endothelial cell surface, perhaps through interaction with P-selectin.^14^ Under fluid shear stress, the UL-VWF strings are cleaved by ADAMTS13 at the Tyr1605-Met1606 bond in the A2 domain^3^ to generate the range of VWF multimer sizes that normally circulate in the blood. VWF serves as the primary adhesive link between platelets and subendothelium and it also carries and stabilizes coagulation factor VIII (FVIII) in the circulation. These hemostatic functions depend upon the ability of VWF to bind circulating factor VIII, subendothelial collagens, platelet glycoprotein Ibα (GPIbα) and integrin αIIbβIII, but the regulation of platelet adhesion depends upon cleavage of VWF multimers by ADAMTS13 (Figure 1).^15^ However, VWF in plasma adopts a folded globular conformation that does not bind to platelet GPIbα and is not cleaved by ADAMTS13.^16^ Fluid shear stress,^17^ or binding to certain surfaces, changes the conformation of VWF so that it assumes an elongated form, disclosing the buried binding site for platelet GPIbα, localized in the A1 domain of the protein. Upon this physically-induced conformational transition, VWF multimers bind tightly to platelet GPIbα and, at the same time, can be recognized by ADAMTS13. A similar modulating effect *in vitro* is achieved by including antibiotic ristocetin or by denaturing reagents such as urea and guanidine-HCl.^2^,^3^,^18^,^19^ The stretched conformer of VWF, more prone to ADAMTS13 proteolysis, is stabilized *in vivo* through the interaction with P-selectin.^20^ Inability to cleave the newly released UL-VWF multimers^1^,^21^,^22^ owing to hereditary or acquired deficiency of plasma ADAMTS13 activity may induce spontaneous VWF-dependent platelet adhesion and aggregation,^23^ leading to disseminated microvascular thrombosis as seen in patients with TTP.

## ADAMTS13 Structure and Function

**Figure 2:**
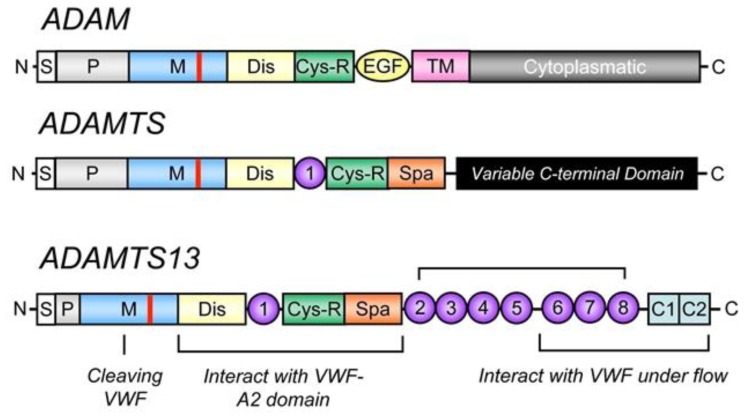
Schematic diagram of ADAM, ADAMTS and ADAMTS13 structure. The structural domains are indicated: signal peptide (S), propeptide (P), metalloprotease (M) (location of zinc-binding motif shown in red), disintegrin domain (Dis), first thrombospondin type 1 (TSP1) repeat (1), cysteine-rich domain (Cys-R), spacer domain (Spa), the second to eighth TSP1 repeats(2) through (8) and two CUB domains (C1 and C2). The metalloprotease domain is the catalytic center that cleaves von Willebrand factor (VWF). The proximal carboxyl-terminal domains from Dis to Spa interact with the A2 domain of VWF. More distal carboxyl-terminal domains (TSP1 2–8) interact with VWF under fluid shear stress. EGF indicates epidermal growth factor-like repeat and TM, transmembrane domain.

The human *ADAMTS13* gene is located on chromosome 9 at position 9q34. It spans 37 kb in length and contains 29 exons.^6^,^8^ ADAMTS13 mRNA is approximately 5 kb and encodes a 1427 amino acid protein. Several alternatively spliced mRNA variants have been characterized; their significance remains unknown.^6^,^8^ The predicted molecular weight of 145 kDa differs from the observed molecular mass of purified plasma ADAMTS13 (~190 kDa),^24^,^25^ and this difference is likely due to its extensive glycosylation.^26^ ADAMTS13 is synthesized predominantly in liver,^6^–^8^,^25^ although variable expression has been observed in endothelial cells,^27^,^28^ endothelial glomerular cells^29^ megakaryocytes or platelets^30^,^31^ and secreted into plasma as an already active enzyme. Mutations in the *ADAMTS13* gene^27^ may result in a reduced or an aberrant secretion of ADAMTS13 protein into the circulation. Various truncated forms of ADAMTS13 are detectable in plasma,^32^ perhaps owing to alternative splicing of ADAMTS13 mRNA or proteolysis of ADAMTS13 by serine proteases such as thrombin^33^ and leukocyte elastase.^34^ Human placenta and skeletal muscle synthesize a 2.4 kb ADAMTS13 mRNA.^8^ There are some evidences from *in vivo*^35^,^36^ and *in vitro*^36^,^37^ studies that ADAMTS13 mRNA and protein are produced in liver hepatic stellate cells. However, the contribution of hepatic stellate cells to plasma levels of ADAMTS13 remains to be determined. Considering the large surface area of vascular endothelial beds, plasma ADAMTS13 might be derived mainly from endothelial cells even though each endothelial cell produces little amounts of ADAMTS13 compared to hepatic stellate cells.^27^,^28^ ADAMTS13 is the 13^th^ member of the ADAMTS family of zinc proteases, which is related to the large ADAM (A Disintegrin And Metalloprotease) family. The ADAMTS family of zinc metalloproteases contains 19 members that share the common structure of a hydrophobic signal sequence, a propeptide, a metalloprotease domain, a thrombospondin type 1 (TSP1) repeat, a disintegrin-like domain, a cysteine-rich domain and a spacer domain.^6^,^8^ In contrast to ADAM proteases, ADAMTSs lack EGF-like repeats and a transmembrane domain and, therefore, are secreted rather than membrane bound enzymes. In addition, all ADAMTS family members possess one or more thrombospondin type 1 (TSP1) motifs^38^ and variable additional C-terminal domains. The carboxyl terminus of ADAMTS13 contains seven more TSP1 repeats and two CUB domains, which are named after motifs first identified in Complement components C1r and C1s, sea urchin protein Uegf and Bone morphogenetic protein-1(Figure 2).^39^

**Figure 3:**
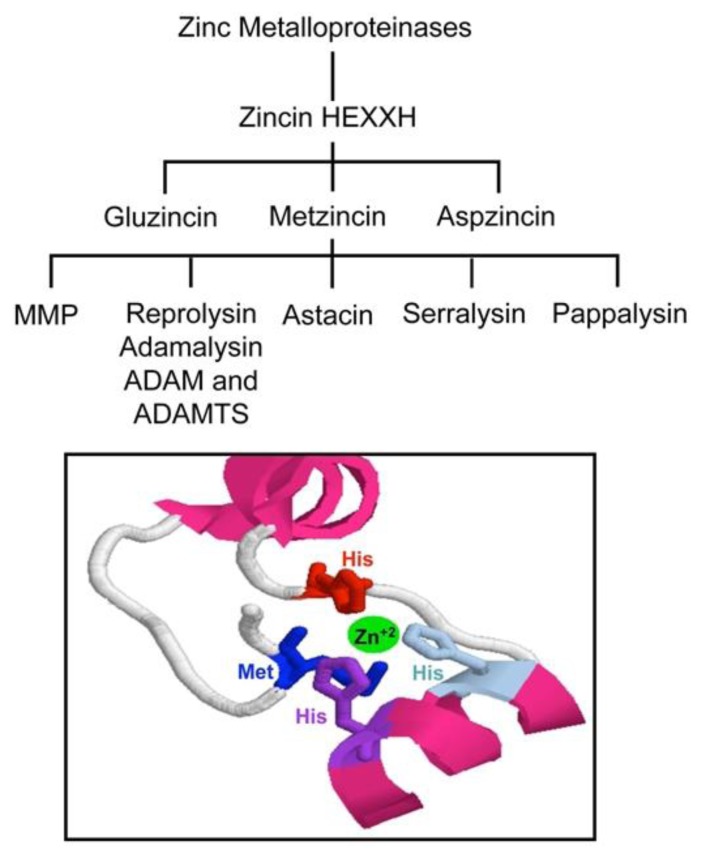
The zinc metalloproteinases of the zincin type that have the minimal catalytic zinc-binding motif containing two histidine residues flanking the catalytic glutamate, HEXXH, comprise three superfamilies: the gluzincins, the aspzincins and the metzincins. Within the metzincins, the major families are the matrixins or matrix metalloproteinases (MMPs), the reprolysins (also known as adamalysins, which includes some ADAM (a disintegrin and metalloproteinase) and ADAMTs (ADAMs with thrombospondin repeats proteins) and the astacins. Metzincins have an HEXXHXXGXXHZ…M motif with three histidine residues binding the zinc ion and an invariant methionine turn in the active site that generates the name metzincins. X represents any amino acid residue and Z indicates a subfamily specific conserved residue, which is D for both ADAM and ADAMTS members. (Inset) Homology modeling of the metalloproteinase (M) domain of ADAMTS-13. The structure was generated using the program RasMol vs. 2.7.5. The structure of the polypeptide chain 80–290, corresponding to the M-domain of ADAMTS13 was modeled by homology on the crystallographic structure of ADAMTS4 solved at 2.80 Å (PDB entry code: 2RJP). Zinc ion (green) is shown together with the three catalytic His-residues. The “Met-turn” typical of the metzincin family is also indicated.

Globally, the family of ADAMTS is composed of enzymes whose main functions include: (1) collagen processing; (2) cleavage of the matrix proteoglycans aggrecan, versican and brevican; (3) inhibition of angiogenesis; and (4) blood coagulation homoeostasis as the von Willebrand factor cleaving protease. Roles in organogenesis, inflammation and fertility are also apparent. Some *ADAMTS* genes have been found to show altered expression in arthritis and various types of cancer. For instance, ADAMTS2 cleaves the propeptide of collagen II, and mutations in this protein are responsible for the Ehlers-Danlos syndrome type VII C.^40^ Mutations in *ADAMTS10* cause autosomal recessive Weill-Marchesani syndrome, a connective tissue disorder characterized by abnormalities of the lens of the eye, proportionate short stature, brachydactyly and joint stiffness.^41^ ADAMTS1, ADAMTS4 and ADAMTS5/11 (also known as aggrecanases) cleave the cartilage proteoglycan aggrecan and may play a role in inflammatory joint disease.^42^–^44^ Interestingly, an anti-inflammatory role has also been recently attributed to ADAMTS13.^45^ Since the isolation and cloning of the ADAMTS13 cDNA, several laboratories have expressed recombinant ADAMTS13 in cell culture. Recombinant ADAMTS13 cleaves VWF *in vitro*, providing a formal demonstration that ADAMTS13 is indeed the VWF-cleaving protease identified in earlier studies.^25^,^26^ The detailed structure of the full-length ADAMTS13 molecule is not yet solved. Only recently, the X-ray diffraction map of the recombinant ADAMTS13 fragment composed of the thrombospondin-1 (TSP-1) type-1 repeat domain (T), the cysteine-rich (C) region, and the spacer domain (S) has been reported.^46^ Very recently, the crystal structure of the P475S mutant of ADAMTS13-DTCS (DTCS-P475S, residues 287–685) was solved and compared with the wild-type structure.^47^ The propeptide of ADAMTS13 contains 41 amino acids, in contrast to the approximately 200 amino acids that comprise the propeptides of most other members of ADAM and ADAMTS family.^48^,^49^ Like other proteases, ADAMTS13 propeptide presents a typical proprotein processing site (RQRR), which has been shown to be a furin cleavage site.^8^ At variance with what has been observed for other metalloproteases, deletion of the ADAMTS13 propeptide does not impair secretion or enzymatic activity, demonstrating that the propeptide is not required for folding or secretion and likely does not confer enzymatic latency.^49^ Moreover, it has been shown that a mutation in the furin consensus recognition site leads to secretion of an active pro-ADAMTS13.^49^ Detection of anti-propeptide antibodies in some patients with TTP suggests that not all plasma ADAMTS13 has this sequence removed.^50^ The metalloprotease domain of ADAMTSs consists of about 200 amino acids. The structural relationship of ADAMTSs to other zinc matrix metalloproteinases (MMPs) is shown in Figure 3. ADAMTSs are reprolysin-like proteins, which, together with ADAMs, MMPs, astacins and serralysins, constitute the metzincin superfamily. The catalytic domains of ADAMTS proteinases share a high degree of similarity and contain the zinc-binding sequence, in which the catalytic Zn2+ ion is coordinated by the three histidine residues, “H224EXXHXXGXXHD235”, where ‘X’ represents any amino acid residue and the conserved aspartic acid residue distinguishes the ADAMs and ADAMTSs from other metalloproteinases. The glutamate following the first zinc-binding histidine has a catalytic role,^51^ polarising a water molecule through hydrogen bonding, which is stabilised by coordination with the Zn2+ ion and is responsible for the nucleophilic attack on the carbonyl of the substrate scissile peptide bond.^52^,^53^,^54^ As in all MMPs and adamalysins, the zinc-binding sequence is followed a short distance from the C-terminal end (10–20 amino acids after the third histidine),^55^ by a conserved methionine residue, an active-site arrangement that has been termed ‘metzincin-type’. This methionine constitutes the ‘Met-turn’, a tight turn arranged as a right-handed screw that seems to serve an important function in the structure of the active site.^53^ It could form indeed a hydrophobic base beneath the catalytic Zn^2+^. Different studies using C-terminal truncations of recombinant ADAMTS13 have shown that the metalloprotease domain alone was not able to cleave plasma VWF.^49^,^56^,^57^

Truncation of ADAMTS13 within or distal to TSP1 results in generation of enzymes that retain VWF-cleaving activity *in vitro*, while truncations proximal to TSP1 (within the protease, TSP1, cysteine-rich or spacer domains) result in an inactive protein. These results indicate that the protease domain alone, even if functional, is not sufficient to recognize and specifically cleave the VWF cleavage site, suggesting that sequences within the region spanning the protease domain to the spacer domain of ADAMTS13 are necessary for VWF-cleaving activity, at least *in vitro*. The mechanistic aspects driving the molecular recognition and cleavage of VWF by ADAMTS13 have been recently unraveled in elegant studies.^33^,^58^ These studies showed that the domains between the metalloprotease and the spacer domain are critical for substrate recognition and cleavage because the mutants lacking one or more of these domains do not cleave multimeric VWF.^57^ Modulation of the ADAMTS13/VWF interaction is critical for an efficient proteolysis and involves both VWF and ADAMTS13. The latter binds to VWF under static conditions and under both venous (2.5 dyn/cm^2^) and arterial (30 dyn/cm^2^) shear stress. This interaction, however, is unproductive for proteolysis unless shear stress is high enough to stretch VWF and expose the buried A2 domain for cleavage.^17^,^59^ Under static conditions, ADAMTS13 cleaves VWF only under denaturing conditions,^2^,^3^ or in the presence of the antibiotic ristocetin,^60^ whereas under conditions of high shear stress found in the microvasculature, VWF proteolysis is extremely rapid and occurs in the absence of any chemical effector.^3^,^17^,^61^ Fluid shear stress alters the conformation of VWF so that the binding and catalysis of ADAMTS13 takes place at the VWF A2 domain.^62^ High shear stress causes micro- and macro-conformational changes in VWF.^63^ These hydrodynamic forces cause conformational changes in VWF that expose a binding site in the A1 domain for the platelet glycoprotein Ib (GPIb) molecule,^64^ facilitating the process of platelet adhesion to the subendothelium. It has to be noted that, once secreted by endothelial cells, UL-VWF is trimmed by ADAMTS13, with production of smaller VWF fragments In the absence of ADAMTS13 activity, either due to genetic mutations or formation of anti-ADAMTS13 autoantibodies, a life-threatening disease, referred to as thrombotic thrombocytopenic purpura (TTP), does occur causing an uncontrolled microvascular thrombosis (see below).^65^ The unique requirement of shear forces, which permit the cleavage by ADAMTS13 of the Tyr1605-Met1606 peptide bond, finely regulates ADAMTS13 activity and impedes an uncontrolled VWF proteolysis from taking place. Moreover, the VWF-cleaving activity may be positively or negatively modulated by the other structural elements of VWF:^66^ heparin sulfate, platelet GPIbα, sodium chloride^60^ and inflammatory cytokines.^67^ Other factors may influence ADAMTS13 and VWF interactions, such as inflammatory cytokines^67^ and hemolysis products.^68^ It cannot be ignored that several Authors have shown that leukocyte proteases such as cathepsin G, elastase, proteinase 3 and MMP9 are able to hydrolyze VWF near or even at the same site where ADAMTS13 proteolyzes the VWF molecule in the A2 domain.^69^ Interestingly, while oxidative modification of VWF Met1606 strongly inhibits proteolysis by ADAMTS13,^70^,^71^ it may even accelerate the cleavage by leukocyte serine proteases.^72^ Recent studies showed the potential of leukocyte zinc- and serine proteases present in thrombi to inhibit the adhesion of VWF to platelets under high shear stress and proved that this phenomenon strictly depends on VWF proteolysis.^73^ This alternative control of VWF function is likely linked to local compartments in blood clots, where the serine proteases are relatively protected against their abundant plasma inhibitors, such as α2-macroglobulin and antithrombin.

## ADAMTS13 and its Role in the Pathogenesis of Thrombotic Microangiopathy, a Pleiomorphic Clinical Setting

**Figure 4:**
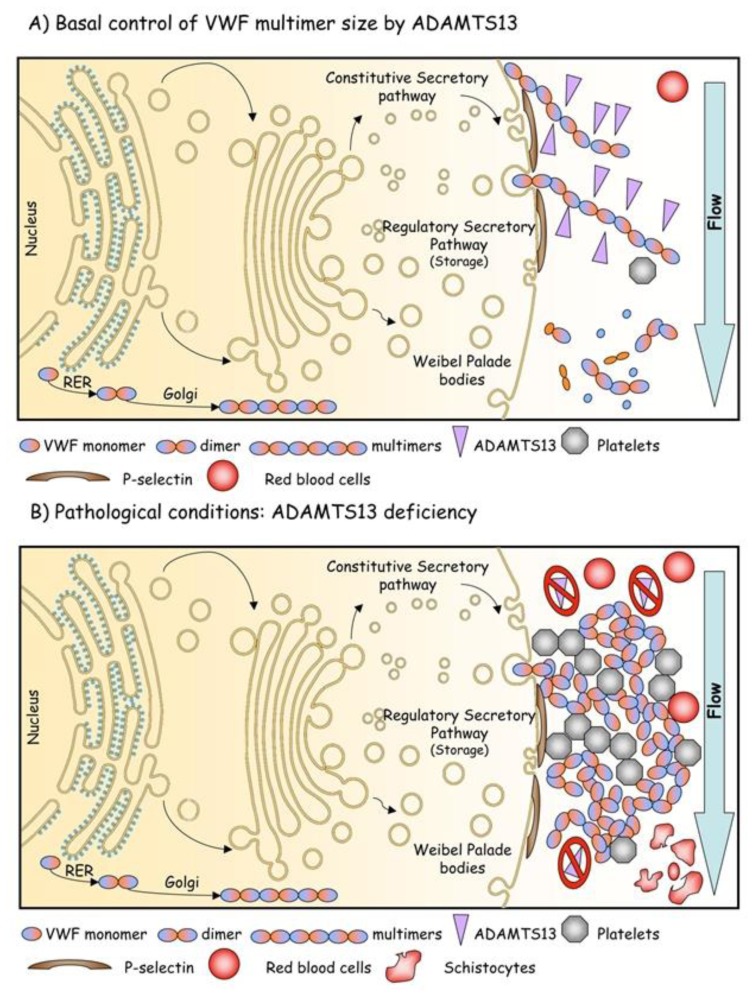
Pathogenesis of TMA caused by ADAMTS13 deficiency. A) Von Willebrand factor (VWF) multimers, produced and stored in the Weibel-Palade bodies of the endothelial cells, are secreted and adhere to endothelial cell membranes via GpIbα and P-selectin. Platelets adhere to VWF multimers through platelet membrane glycoprotein GPIbα. In flowing blood under high shear stress, VWF in the platelet-rich thrombus is in a stretched conformation and is trimmed by ADAMTS13, which limits thrombus growth. B) If ADAMTS13 is absent or inhibited by autoantibodies, VWF-dependent platelet accumulation is uncontrolled and may cause microvascular thrombosis, formation of schistocytes and, ultimately, TMA.

Thrombotic macroangiopathies (TMAs) refer to the disorder of diffuse microvascular thrombosis involving the capillary and arteriolary bed of the brain, kidney and other organs. The patients typically present with 1) severe thrombocytopenia (<50,000 plts/μl), 2) non-immune hemolysis with presence of schistocytes on blood smears and 3) variable neurologic abnormalities reaching even coma and/or acute renal failure.^74^ Thrombocytopenia results from peripheral consumption of platelets in the microvasculature, whereas erythrocyte fragmentation and hemolysis stem from mechanical injury induced by passage of erythrocytes through platelet thrombi under abnormally high shear stress in the microvasculature (Figure 4). TMAs area group of severe clinical settings that, without treatment, undertake a rapid worsening and death in most cases. Plasma exchange or infusion is the mainstay of treatment for most TMAs. As anticipated above, the pathogenesis of complex syndromes such as TMAs is mostly explicable on the basis of the deficiency of ADAMTS13. However, it should be noted that TMAs are not monogenetic diseases. Thus, the clinical manifestations of this group of disorders are highly variable and heavily affected by the co-existence of other genetic and environmental modifiers. This group of TMAs is constituted by different clinical settings referred to as Thrombotic Thrombocytopenic Purpura (TTP), hemolytic uremic syndrome (HUS), diarrhea-associated HUS or atypical HUS. Unfortunately, any existing clinical or pathological classification of TMAs is based on assumptions that have never been validated. The greatest uncertainty has involved deciding whether certain cases represent examples of TTP or HUS. A rule of thumb has suggested that HUS may usually be distinguished from TTP because HUS occurs predominantly in individuals younger than 10 years, while TTP occurs predominantly in adults. However, this differentiation is not reliable, as either condition can occur in either group. Other clinical features aid in distinguishing the conditions at any age of onset. For instance, renal manifestations are usually more prominent in HUS than neurological ones, whereas neurological manifestations are usually more prominent in TTP than renal ones. Fever precedes TTP more commonly than it precedes HUS.^75^ Despite these distinctions, continued recognition of borderline or atypical cases has generated doubts about the possibility that objective criteria other than age are able to distinguish “atypical” HUS from “atypical” TTP. This problem led to the application of the unsatisfactory term TTP-HUS to mean an indistinctly defined and clinically heterogenous collection of cases between classic TTP and classic HUS. The recognition of phenotypic instability in recurrent cases encouraged use of this term. For example, 1 patient had 5 episodes manifesting the HUS phenotype before the age of 15 years and 9 episodes manifesting the TTP phenotype after 20 years of age.^76^

It should be noted that TTP and HUS share the fundamental pathologic feature of arteriolar thrombosis with vessel wall intimal swelling and fibrinoid necrosis. However, the composition of the thrombi differs histopathologically, at least in well-defined cases of TTP and HUS. Those of such well-defined TTP cases contain degranulated platelets and von Willebrand factor. Those of Shiga toxin–provoked HUS are rich in fibrin and thus arise from activation of the plasma coagulation cascade.^4^

Fortunately, recent advances in understanding the pathogenesis of TTP somewhat clarified the boundaries between microangiopathic disorders with renal or neurological manifestations, and they have produced useful diagnostic tests for some forms of clinically defined TTP.

### A) Relationship Between ADAMTS13 and Occurrence of TMAs

Investigations have demonstrated a high degree of relevance in the relationship of ADAMTS13 to TTP. These investigations defined a heritable form of TTP with severe (<5%) ADAMTS13 activity deficiency and an acquired form due to the elaboration of antibodies directed at 1 or more ADAMTS13 epitopes.^77^ However, many thrombotic microangiopathies (TMAs) are not associated either with severe ADAMTS13 activity deficiency or with antibodies that block ADAMTS13 activity. This class of patients may represent >30% of all TMA patients.^16^ In some instances, the clinical syndrome is indistinguishable from typical TTP. At autopsy, widespread hyaline thrombi, accompanied by variable fibroblastic infiltration and endothelial overlay, are found in the terminal arterioles and capillaries of multiple organs. The thrombi are found most extensively in the heart, brain, kidney, pancreas, spleen, mesentery and adrenal gland, and are composed primarily of platelets and von Willebrand factor.^78^–^80^ A small amount of fibrin may be present surrounding the amorphous or granular materials. In older lesions, hyaline deposits may be seen in the sub-endothelial layers of capillaries and between the endothelium and muscular layers of arterioles. Pre-occlusive pseudoaneurysmal dilatation may also be present. Fibrinoid necrosis and vascular or perivascular inflammatory cell infiltration are characteristically absent or minimal. Some cases, especially those in adults, are associated with promoting factors that are associated with the development of typical hereditary or acquired TTP. Recent schemes have used the identification of such promoting factors to classify TTP-like thrombotic disorders without severe or acquired abnormalities of ADAMTS13 function, as just defined. These entities tend to occur in adults and sometimes manifest features that occur along a clinical spectrum between TTP and HUS. Many of these illnesses cannot be distinguished by using currently available laboratory tests, except when the underlying etiologic illnesses are symptomatic. These conditions share with TTP and HUS the fundamental finding of thrombocytopenic and hemolytic TMA on peripheral blood smear. In the following paragraphs, we will treat only the ADAMTS13-related forms of TMAs/TTP. These syndromes include: 1) the congenital and 2) acquired deficiency of the metalloprotease. Finally, we will mention a recently discovered pathogenetic mechansisms that can be responsible for accumulation of UL-VWF multimers and promote forms of TMAs in cardiovascular and metabolic disorders by perturbing the VWF/ADAMTS13 interaction.

### B) Congenital ADAMTS13 Deficiency

**Figure 5:**
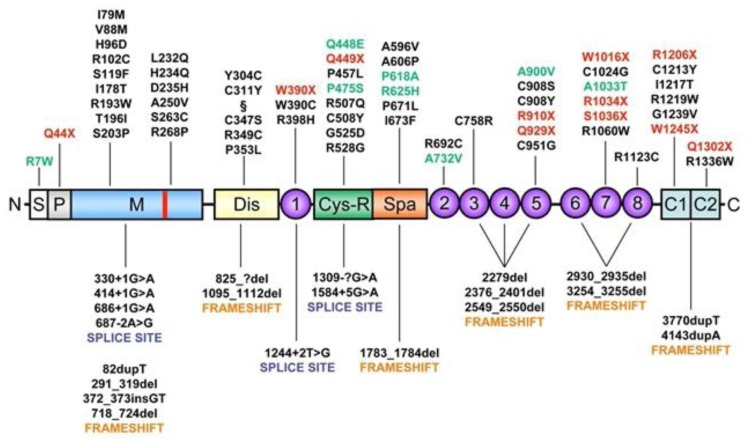
Linear map of the location of ADAMTS13 mutations found in patients with congenital thrombotic thrombocytopenic purpura (TTP) [Upshaw-Schulman syndrome (USS)]. The missense mutations, nonsense mutations (red) and single nucleotide polymorphisms (SNPs) (green) are shown above the domain structure of ADAMTS13. The mutations that result in alternative splicing of ADAMTS13 mRNA or frameshifts are listed under the domain structure of ADAMTS13. S indicates the signal peptide; P, propeptide; M, metalloprotease (location of zinc-binding motif shown in red); Dis, disintegrin domain; 1, first thrombospondin type 1 (TSP1) repeat; Cys-R, cysteine-rich domain; Spa, spacer domain; 2 through 8, the second to eighth TSP1 repeats; C1 and C2, two CUB domains (for complement C1r/C1s, Uegf, Bmp1 domain) §p.[C322G (+) T323R (+) F324L].

Many studies in different ethnic populations have demonstrated the presence of ADAMTS13 mutations in patients with TTP.^16^,^26^,^48^,^68^,^81^–^106^ Some aspects emerging from studies of ADAMTS13 congenital deficiency in mice could help to unravel the role of ADAMTS13 and UL-VWF multimers in the pathogenesis of TMAs. For instance, inactivation of the *ADAMTS13* gene in mice failed to generate the phenotype of TTP microvascular thrombosis until the ADAMTS13 null allele was transferred to a particular mouse strain, CASA/Rk, that has increased levels of VWF.^107^,^108^ Nevertheless, cross-breeding studies showed that the development of TTP is independent of mouse plasma VWF levels. In CASA/Rk mice with homozygous ADAMTS13 null alleles, spontaneous thrombosis and death occur in post-neonatal life.^107^ Only administration of shiga toxin is able to induce a massive secretion of UL-VWF multimers from endothelial cells.^109^ From a clinical standpoint, there is no evidence of antecedent shiga toxin exposure in patients of TTP. Only a small fraction of TTP patients has elevated plasma VWF levels. Thus, the relevance of the shiga toxin-ADAMTS13-deficient mouse model to either TTP or shiga toxin-associated HUS remains uncertain. We can only speculate that the lack of a thrombotic phenotype in some mouse strains with severe deficiency of ADAMTS13 due to its gene inactivation suggests in these strains the presence of modifiers that affect the response of VWF to shear stress. To date, about 80 mutations responsible for hereditary TTP have been identified in the *ADAMTS13* gene.^16^,^26^,^48^,^68^,^81^–^106^ Seven are splice mutations, ten frameshift deletions, four frameshift insertions, eleven nonsense mutations and the remaining 45 mutations lead to codon changes. Moreover, numerous Single Nucleotide Polymorphisms (SNPs) have been recognized in recent years: eight of these SNPs are expressed and affect expression, secretion and activity of the enzyme, whereas eighteen are silent. The mutated sites in *ADAMTS13* are distributed across many exons and introns throughout the gene. The absence of clusters (“hot-spots”) of mutations within the metalloprotease domain implies structural and functional importance of other regions besides the catalytic site. This finding is in-line with the observed relevance of exosites in ADAMTS13 in the molecular recognition and proteolytic processing of VWF^49^,^64^,^110^ under both static and high shear rate conditions (see above). In patients with hereditary TTP, homozygous or compound heterozygous mutations of the *ADAMTS13* gene lead to severe ADAMTS13 deficiency. Globally, the affected residues span the entire spectrum of the *ADAMTS13* gene. Figure 5 shows the principal mutations discovered along the *ADAMTS13* gene. Mutations of the *ADAMTS13* gene may cause impaired protein synthesis, secretion or proteolytic activity, depending on its localization, as determined in numerous site-directed mutagenesis and expression studies. Heterozygous individuals have ADAMTS13 activity at 40–70% of normal values, while a TTP phenotype is present in more than 90% of the patients with double heterozygous or homozygous mutations. However, it may be predicted that variable phenotypic severity of TTP may arise from the various *ADAMTS13* mutations.^111^ Only a few mutations have been described in more than one pedigree. A notable exception is 4143dupA, which has been described in multiple pedigrees of Northern and Central Europe and in Turkey. Haplotype analysis suggests that many, if not all, of the 4143dupA mutant alleles probably originated from a common ancestry.^87^ Why this particular allele is much more frequent than other mutant alleles remains an unanswered question. Other ethnical characteristics concern one ADAMTS13 variant allele, 1423C>T (P475S), found in Japanese (5.1%), Koreans (4%) and Chinese (0.5–1.7%) but not detected among Caucasians or African Americans.^26^,^112^ This polymorphism had raised considerable interest because in expression studies this mutation markedly reduces the activity of ADAMTS13 to approximately 10% of control, raising the possibility that partial deficiency of ADAMTS13 deficiency may be quite common among Northeast Asians. Nevertheless, this prediction was not correct, as more recent investigations have shown that carriers of the P475S polymorphism have only a minor decrease (10%) of the ADAMTS13 activity and revealed that the previously reported low activity of the P475S variant resulted from the effect of high urea concentration used in the ADAMTS13 activity assay.^113^ Thus, this mutant might have only an abnormal stability. Recently, a novel mutation causing a severe ADAMTS13 deficiency, p.E735X, has been detected in a 2 year old Tunisian child presented with chronic thrombocytopenic purpura, which failed to respond to corticosteroids.^114^

### C) Inhibitors of ADAMTS13

A strong deficiency of ADAMTS13 activity can also be associated to development of auto-antibodies against the protease. The formation of IgG or IgM anti-ADAMTS13 antibodies may be responsible for the onset of TMAs idiopathic or secondary to drugs, pregnancy or diseases such as infections, cancers and autoimmune diseases.^21^,^115^ In patients with acquired TTP, deficiency of ADAMTS13 results from autoimmune inhibitors of ADAMTS13, which either inhibit its catalytic activity or induce a rapid clearance from the circulation.^16^,^116^–^121^ Similar to other autoimmune disorders, the etiologies of acquired TTP are unknown and TTP patients often exhibit positive autoimmune reactions to different target antigens [], 117 suggesting that defective immune regulation may contribute to the development of TTP. A defective regulation of T-reg and tolerogenic dendritic cells may be responsible for the occurrence of anti-ADAMTS13 antibodies, in analogy with what has been shown in other autoimmune coagulation inhibitors, such as anti-FVIII antibodies.^122^ HIV infection may also be a risk factor for TTP, although this association has not been confirmed by all Authors.^123^ The inhibitors are more frequently IgG, although occasional production of IgA and IgM antibodies has been described. In a recent study, IgG(4) was found to be the most prevalent IgG subclass (90%) in 58 patients with acquired TTP, followed by IgG(1) (52%), IgG(2) (50%) and IgG(3) (33%).^124^ These studies also showed that IgG(4) may be found either alone (33%) or with other IgG subclasses (67%).^124^ IgG(4) was not detected in 10% of the patients. Patients with high IgG(4) levels and undetectable IgG(1) are more prone to relapse than patients with low IgG(4) levels and detectable IgG(1) []. 124 Remarkably, a rising ADAMTS13 inhibitor level may be associated with switching of the IgG subclasses, suggesting that cytokine dysregulation may be responsible for the rising inhibitor levels observed in some cases of TTP.^125^ Epitope mapping studies showed that the spacer domain,^33^,^50^,^126^ specifically residues T572-N579 and V657-G666,^33^ comprise a common antigenic core region that is a relevant target for ADAMTS13 antibodies in TTP. Notably, the proteolytic activity of ADAMTS13 variants truncated upstream of the Cys-rich domain is not generally inhibited by the inhibitors of patients with TMAs. These non-inhibited ADAMTS13 recombinant constructs may be used to overcome, at least in part, the difficult management of patients with high inhibitor levels. The levels of the ADAMTS13 inhibitors tend to be low (<10 U/mL),^118^,^127^ often receding to even lower or undetectable levels within weeks or months. Such characteristics of the ADAMTS13 inhibitors suggest that the immune response is induced by exposure to exogenous antigens with molecular mimicry to ADAMTS13. The level of anti-ADAMTS13 inhibitors determines the efficacy of therapeutic strategies, particularly plasma exchange, aimed at eliminating their pathologic effects. Usually, ADAMTS13 inhibitors, measured by the Bethesda assay in clinical laboratories,^128^ have low titers (<10 Bethesda units/ml) and self-limited course. However, if the level of anti-ADAMTS13 inhibitors is high, the treatment may fail.^125^ Moreover, refractory TMA forms, characterized by persistent anti-ADAMTS13 inhibitors, have also been reported in patients requiring long-term plasma exchange treatment and immunosuppressive therapy with rituximab.^129^

## The Role of VWF-ADAMTS13 Interaction in Other Arterial Thrombotic Diseases

It has been suggested that VWF plays an important role in the pathogenesis of arterial thrombotic disorders. Previous studies have shown the relevance of platelets and VWF in the initiation of atherosclerotic plaque formation. Both inactivation of VWF and inhibition of VWF-GP1b interaction delay the formation of fatty streaks VWF. From a biological standpoint, it is likely that VWF contributes to the pathogenesis of early atherosclerotic lesions. Hence, many studies have investigated the association between VWF plasma levels and the subsequent risk of cardiovascular disease. In the ARIC study, the relative risk (RR) for coronary artery disease (CHD) for the highest vs. the lowest tertiles of VWF levels was approximately 1.3.^130^,^131^ Moreover, VWF was found to play a relevant role in thrombotic microangiopathies occurring in diabetes mellitus.^132^ More recently, compelling evidence has emerged about the association of high VWF levels with occurrence of ischemic stroke, particularly in the cardioembolic and cryptogenetic subtypes.^133^,^134^ Recently a relative inhibition of VWF-ADAMTS13 interaction linked to oxidative modification of VWF in some clinical settings such as diabetes mellitus and end stage renal disease has been shown to be strongly associated to enhanced incidence of thrombotic macro- and microangiopathies.^71^,^135^,^136^

## Future Directions

After the discovery that normal plasma contains a zinc protease able to specifically proteolyze VWF, the past decade has witnessed the most exciting advances in the history of studies on the pathogenesis of TMAs. However, many issues still need to be addressed. The knowledge of some mechanistic aspects of ADAMTS13 catalysis and its regulation, the development of sensitive and reliable assays in the clinical diagnostics of TMAs and the nature of modifiers of ADAMTS13 activity on VWF multimers in patients affected by TMAs require further improvement. From a biotechnological standpoint, industrial production of partially deleted ADAMTS13, non-suppressible by pathological auto-antibodies, may circumvent the difficulties that replacement therapies with recombinant full-length ADAMTS13 may encounter in patients with acquired TTP. Finally, basic research to clarify the immunological mechanisms of generation of ADAMTS13 inhibitors^137^ will aid in the discovery of new strategies able to improve the prevention, diagnosis and management of TMAs.
